# COVID-19 related acute genital ulcer: a case report

**DOI:** 10.31744/einstein_journal/2022RC6541

**Published:** 2022-01-27

**Authors:** Cláudia Márcia de Azevedo Jacyntho, Marcela Ignacchiti Lacerda, Mariana de Sousa Ribeiro de Carvalho, Maria Roberta Meneguetti Seravali Ramos, Pedro Vieira-Baptista, Sandra Helena de Azevedo Durães Bandeira

**Affiliations:** 1 Hospital Federal dos Servidores do Estado Rio de Janeiro RJ Brazil Hospital Federal dos Servidores do Estado, Rio de Janeiro, RJ, Brazil.; 2 Hospital Universitário Pedro Ernesto Rio de Janeiro RJ Brazil Hospital Universitário Pedro Ernesto, Rio de Janeiro, RJ, Brazil.; 3 Universidade do Estado do Pará Belém PA Brazil Universidade do Estado do Pará, Belém, PA, Brazil.; 4 Fundação Oswaldo Cruz Rio de Janeiro RJ Brazil Fundação Oswaldo Cruz, Rio de Janeiro, RJ, Brazil.; 5 Hospital Lusíadas Porto Portugal Hospital Lusíadas, Porto, Portugal.; 6 Centro de Saúde Dr. Osvaldo Assunção Tucano BA Brazil Centro de Saúde Dr. Osvaldo Assunção, Tucano, BA, Brazil.

**Keywords:** Vulvar diseases, Coronavirus infections, COVID-19, Lipschütz’s ulcer

## Abstract

Acute vulvar ulcer (Lipschütz’s ulcer) is a rare lesion with local hyperimmunoreactivity triggered by infection, which is characterized by acute, painful, and necrotic ulcerations. This condition is usually found in non-sexually active adolescents, and it resolves spontaneously. We report a case of a 35-year-old woman who was diagnosed with COVID-19 who did not have severe symptoms, but had high levels of D-dimer for 9 days. The COVID-19 diagnosis was followed by the appearance of an acute, necrotic, extremely painful vulvar ulcer, although symptoms caused by COVID-19 had improved. We emphasize the importance of the differential diagnosis to exclude diseases such as Behçet’s syndrome, Sexually Transmitted Infections, as well as the presence of viruses that generally trigger Lipschütz’s ulcer, such as Epstein-Barr virus and cytomegalovirus. No treatment is usually necessary, however, in the present report due to the pain experienced by the patient, we successfully used oral prednisone.

## INTRODUCTION

Lipschütz’s ulcers, *ulcus vulvae acutum*, are a non-sexually acquired condition, characterized by the sudden onset of painful and necrotic genital ulcers. Self-resolution without scarring is the usual course, and the Lipschütz’s ulcers may be triggered by Epstein-Barr virus (EBV), cytomegalovirus (CMV), *Mycoplasma pneumoniae* and *Toxoplasma gondii*.^(1)^The diagnosis is of exclusion after ruling out Sexually Transmitted Infections (STIs), idiopathic aphthosis, Behçet’s disease, and extra-genital Crohn’s disease.^(2)^

Recently, two cases associated with severe acute respiratory syndrome coronavirus 2 (SARS-CoV-2) infection have been described in the literature.^(3,4)^ We report a case of a woman with acute vulvar ulcer possibly associated with SARS-CoV-2.

The aim of this case study was to highlight the possible association of Lipschütz’s ulcers with coronavirus disease 2019 (COVID-19).

## CASE REPORT

Woman, 35-year-old, married, unique partner in her life, without intercourse during the previous months, health care professional, non-smoker, no personal or family history of autoimmune diseases, and who denied regular use of medication.

The woman had been diagnosed 9-days before with COVID-19 (positive reverse transcriptase polymerase chain reaction – RT-PCR – nasopharyngeal swab test for SARS-CoV-2) and presented dry cough, fever, headache and myalgia; arthralgia was absent. Criteria of severe disease were absent. During the 72 hours prior to consultation, she had progressive vulvar pain that was self-treated using oral analgesics and topical acyclovir for 2 days. There were no genital or oral ulcers, nor history of intestinal symptoms. Computed tomography chest scan was normal, and blood tests showed normal cell counts, biochemical parameters, and levels of complement C3 and C4, however, D-dimer levels were increased (1,332mcg/L).

**Figure 1:**
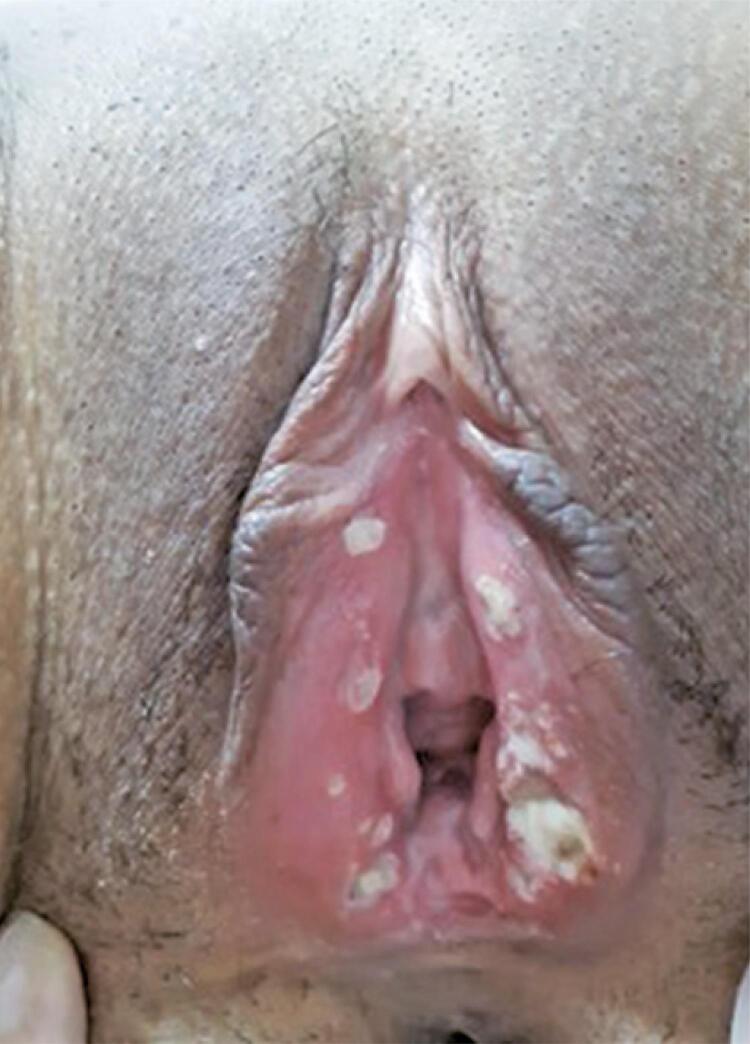
Necrotic and fibrinous vulvar ulcers

On physical examination, we observed partially symmetric bilateral necrotic, extremely painful ulcers with a central fibrinous area that extended to the labia minora (Figure 1). There were no cervical or vaginal abnormalities, cutaneous involvement, oral or anal aphthae, inguinal lymphadenopathy, or fever. Ophthalmologic evaluation showed no abnormalities.

The most common infection causes of genital ulcers were discarded after negative results in serologic tests for Herpes simplex virus (HSV), 1/2 positive immunoglobulin G and M (IgG+/IgM-), *Mycoplasma pneumonae* ( IgM-/IgG-) hepatitis B and C, HIV, EBV, CMV, FTA-ABS and toxoplasmosis. Ulcer scraping for PCR herpes virus testing was not performed due to the local pain and the low suspicion after anamnesis and genital examination.

**Figure 2:**
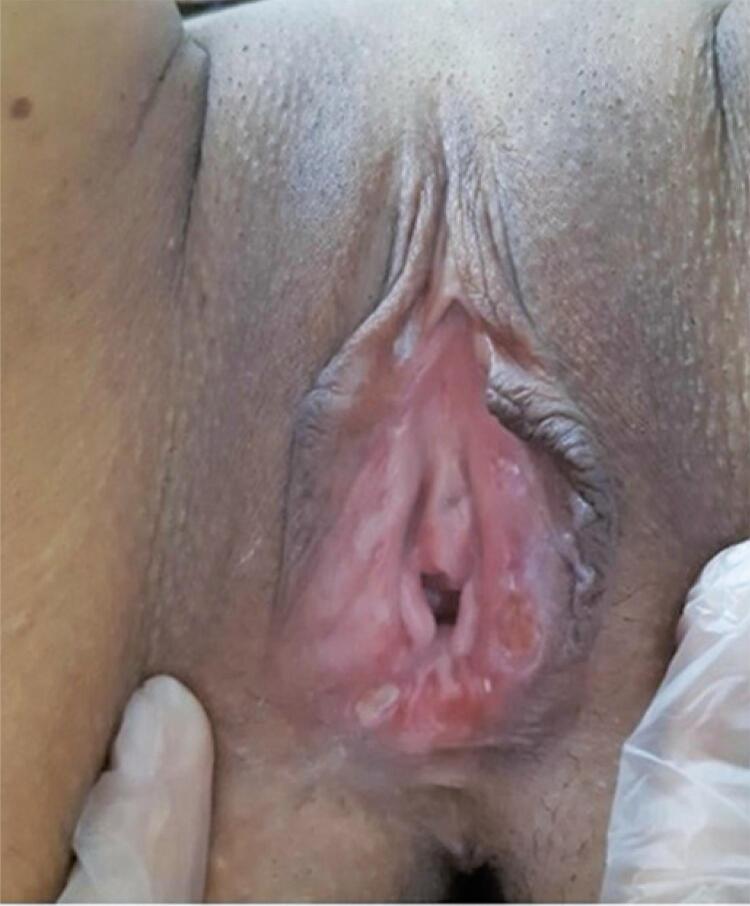
Healed ulcers

Lipschütz’s ulcers related to COVID-19 was considered, after exclusion of Behçet’s disease by applying the International Criteria for Behçet´s Disease (ICBD),^(5)^ as well as due to the other causes of vulvar ulcers outlined above through clinical examination and laboratory tests. Due to the patient’s complaints of progressive pain, we initiated the oral 40mg prednisone daily for 5 days. One week later, the woman had significant improvement of the lesions (Figure 2), as well as pain reduction and complete remission of headache and myalgia. At this time, the SARS-CoV-2 RT-PCR (nasopharyngeal) test was negative and D-dimer levels were normal (430mcg/L). No antibiotic was required.

A final diagnosis of Lipschütz’s ulcers related to COVID-19 was made after excluding other common triggers for Lipschütz’s ulcers, other more specific causes such as Crohn’s disease and STDs, and Behçet’s disease.

This study was approved by the Ethics Committee of *Hospital Federal Cardoso Fontes* (approval number: 4,310,153, CAAE: 38673320.3.0000.8066).

## DISCUSSION

We report a case of a woman with COVID-19 with sudden, painful, necrotic ulcers, which resolved in few days, which was characteristic of a Lipschütz’s ulcers^(2)^ associated with SARS-CoV-2 as the possible trigger of this condition.

We excluded Behçet’s disease, characterized by oral and genital ulcers, ocular lesions, skin lesions, arthritis and occasional neurological involvement.^(5,6)^ The important factor to exclude Behçet’s disease and assuming the case as Lipschütz’s ulcers triggered by COVID-19 was the sudden onset of ulcers with resolution within few days. Moreover, the lack of cutaneous lesions and scars, with vestibular involvement only, favoured the hypothesis of Lipschütz’s ulcers.^(2)^ In our case, biopsies were not necessary due to the rapid resolution.

Lipschütz’s ulcers is considered to affect mostly non-sexually active adolescents.^(2,7)^ The other published cases of vulvar ulcer associated with COVID-19 affected a 41-year-old and 19-year-old women.^(3,4)^ Neither women had previous history of vulvar or vaginal ulcers,^(3,4)^ however oral ulcers were presented in the first case described.^(3)^ The absence of oral aphthae in our report reinforces the diagnosis of Lipschütz’s ulcers. Similarly, to what we described, in the first published case,^(3)^ the woman presented vulvar ulcers after 7 to 10 days of SARS-CoV-2 infection diagnosis and had no severe symptoms or needed hospitalization. In the other case, vulvar involvement was more precocious.^(4)^

Other infections were ruled out in all cases, although previous cases did not report D-dimer values, which were increased in our case. The D-dimer increase in COVID-19 infection has been associated with higher morbidity, despite the scarcity of studies correlating this infection with severity in outpatients. Elevated D-dimer levels indicate a hypercoagulable state and secondary fibrinolysis, which may result in thrombotic disease.^(8)^

The pathophysiology of Lipschütz’s ulcers is not fully understood, however, it is believed that an infection (viral or bacterial) leads to a state of hypersensitivity and subsequent intravascular deposit of immune mediated complexes, formation of microthrombi and tissue necrosis, thus leading to the development of genital ulcers.^(7-9)^

A systematic review of Lipschütz’s ulcers concluded that this condition more frequently affects sexually inactive subjects <20 years of age (a concept that we challenge),^(7)^ who present painful necrotic ulcers that resolve within 3 weeks and had no recurrences as in our case. In addition, Lipschütz’s ulcers is normally associated with infections (88%) and the most frequent is flu-like illness, which in this case was COVID-19, or infectious mononucleosis syndrome.^(2,10)^

## CONCLUSION

This case represents a rare situation of a genital ulcer associated to COVID-19 with elevated D-dimers. We emphasize the importance of differential diagnosis for Lipschütz’s ulcers triggered by SARS-CoV-2, given that there are no strict criteria for its diagnosis*,* as it remains one diagnosis of exclusion.
